# Bacillary Angiomatosis in a Patient With HIV and Disseminated Mycobacterium avium Complex Infection

**DOI:** 10.7759/cureus.63392

**Published:** 2024-06-28

**Authors:** Ibeth P Caceres, Angelique Ruml, Rubi Montejano, Omid Jalali, Theodore Rosen

**Affiliations:** 1 Dermatology, Baylor College of Medicine, Houston, USA; 2 Dermatology, Michael E. DeBakey Veterans Affairs Medical Center, Baylor College of Medicine, Houston, USA

**Keywords:** acquired immunodeficiency syndrome, hiv, mycobacterium avium complex, bartonella henselae, bacillary angiomatosis

## Abstract

*Bartonella *is a genus of arthropod-borne bacterial pathogens that typically cause persistent infections of erythrocytes and endothelial cells in mammalian hosts. The species that primarily infect humans are* Bartonella henselae* and *Bartonella quintana.* Depending on immune status, the clinical presentation of *B. henselae* may differ, manifesting as cat-scratch disease in immunocompetent individuals or bacillary angiomatosis (BA) and peliosis in immunocompromised patients. The cutaneous manifestations of BA are typically characterized by occasionally painful, angiomatous papules and nodules, often with a chronic, persistent course. Herein, we present a case of biopsy-confirmed *B. henselae* infection in a 32-year-old HIV-positive female with acquired immunodeficiency syndrome in the setting of disseminated* Mycobacterium avium* complex infection, an association that has been less frequently described. This case serves as an important reminder to consider uncommon opportunistic infectious etiologies when examining immunocompromised patients, as prompt diagnosis and treatment are essential in this patient population.

## Introduction

First described in 1983, bacillary angiomatosis (BA), also known as epithelioid angiomatosis, is an infectious vascular proliferative disorder affecting the skin and visceral organs, classically manifesting as cutaneous red or violaceous papules and nodules [1]. BA arises from infection with *Bartonella* species, vasculotropic pathogens infecting endothelial cells and erythrocytes [2,3]. Clinically, BA may resemble the malignant lesions of Kaposi sarcoma (KS) or benign lesions such as pyogenic granulomas (PG), verruga peruana, angiokeratomas, and cherry angiomas [1,4,5].

The fastidious Gram-negative bacilli, *Bartonella henselae* and *Bartonella quintana*, have been identified as the causative organisms of BA. *B. henselae* is linked to cat and flea exposure, while *B. quintana* appears most often in individuals with increased exposure to body lice, such as people experiencing homelessness [1]. *B. henselae *and *B. quintana *are responsible for other opportunistic infections besides BA in immunocompromised individuals, such as peliosis and bacteremia [6]. *B. quintana* demonstrates a predilection for subcutaneous and lytic bone lesions, while *B. henselae* is associated with bacillary peliosis of the liver and spleen, but both are known to cause BA and endocarditis [2,7].

Species-specific diagnosis often requires identification using molecular techniques [8]. Isolating *Bartonella* spp. through culture is challenging due to their fastidious and slow-growing nature, requiring special techniques and varying incubation periods of up to five to six weeks [8]. Once cultured, the morphology of *Bartonella* colonies varies based on species. *B. henselae* colonies are uniformly sized, rough-textured, and elevated with pitting of the agar [7]. In contrast,* B. quintana* colonies are heterogeneously sized, smooth, and flat without pitting of the agar [7]. Further, *B. henselae* and *B. quintana* cannot be easily differentiated using serological testing. Immunological testing with an indirect immunofluorescence assay (IFA) or enzyme immunoassay demonstrates serologic cross-reactivity between both species [9]. Therefore, while serological testing can support the diagnosis of infection with *Bartonella* spp., it is not reliable for species-specific diagnosis. Additionally, IFA has not been well-validated for individuals with HIV [10].

In humans, infection of the endothelium with *B. henselae* can lead to marked angiogenesis, which manifests clinically in the formation of benign vascular tumors [3]. *Bartonella*-triggered vascular tumor formation is reminiscent of tumor angiogenesis, a complex sequence of morphogenetic events including pro-angiogenic factors such as vascular endothelial growth factor that result in disordered, immature blood vessel formation that sprouts from pre-existing vessels. Vascular colonization by the microorganisms is followed by a nuclear factor kappa B-mediated proinflammatory response that subsequently inhibits endothelial cell apoptosis and directly stimulates endothelial cell proliferation and differentiation [3]. The growth of *Bartonella*-triggered vascular tumors depends on the continuous presence of bacteria in the lesion, as is typically observed in chronically infected immunosuppressed patients [3].

Although BA has been described to occur in immunocompetent individuals, it predominantly manifests in immunocompromised individuals, especially in patients with HIV and acquired immunodeficiency syndrome (AIDS) presenting with CD4 counts less than 100/mm^3^ [11,12]. In the era of effective antiretroviral therapy (ART), the current incidence of BA is difficult to establish; however, previous studies have suggested an incidence of 0.1% among individuals with HIV [1]. We present a classic case of BA in a treatment-naive HIV-positive patient in the setting of coinfection with disseminated *Mycobacterium avium* complex (MAC).

## Case presentation

A 32-year-old female with a history of treatment-naive HIV for over a decade, due to unclear reasons, presented for initial hospitalization for recurrent fevers associated with generalized weakness, nausea, and vomiting. A physical exam uncovered a petechial rash in the extremities. Blood work was notable for pancytopenia, with a CD4 count of 4/mm^3^ and a viral load of 7.86 x 10^5^ copies/mL. Imaging revealed diffuse lymphadenopathy and hepatosplenomegaly. Given multiple subcentimeter nodular lesions in the liver, bacillary peliosis was a concern. Despite an extensive infectious workup, the results for cryptococcal antigen, urine histoplasma antigen, coccidioides antibody, *Bartonella* spp. antibodies, rapid plasma reagin, cerebrospinal fluid studies, cytomegalovirus PCR, Epstein-Barr virus PCR, and urine and blood cultures were unrevealing. A lymph node biopsy showed reactive lymphoplasmacytic proliferation. The patient was stabilized and discharged empirically on doxycycline to cover for *Bartonella* and trimethoprim-sulfamethoxazole for *Pneumocystis jirovecii* pneumonia (PJP) prophylaxis with outpatient infectious disease follow-up to commence ART. The patient successfully completed approximately six weeks of ART.

**Figure 1:**
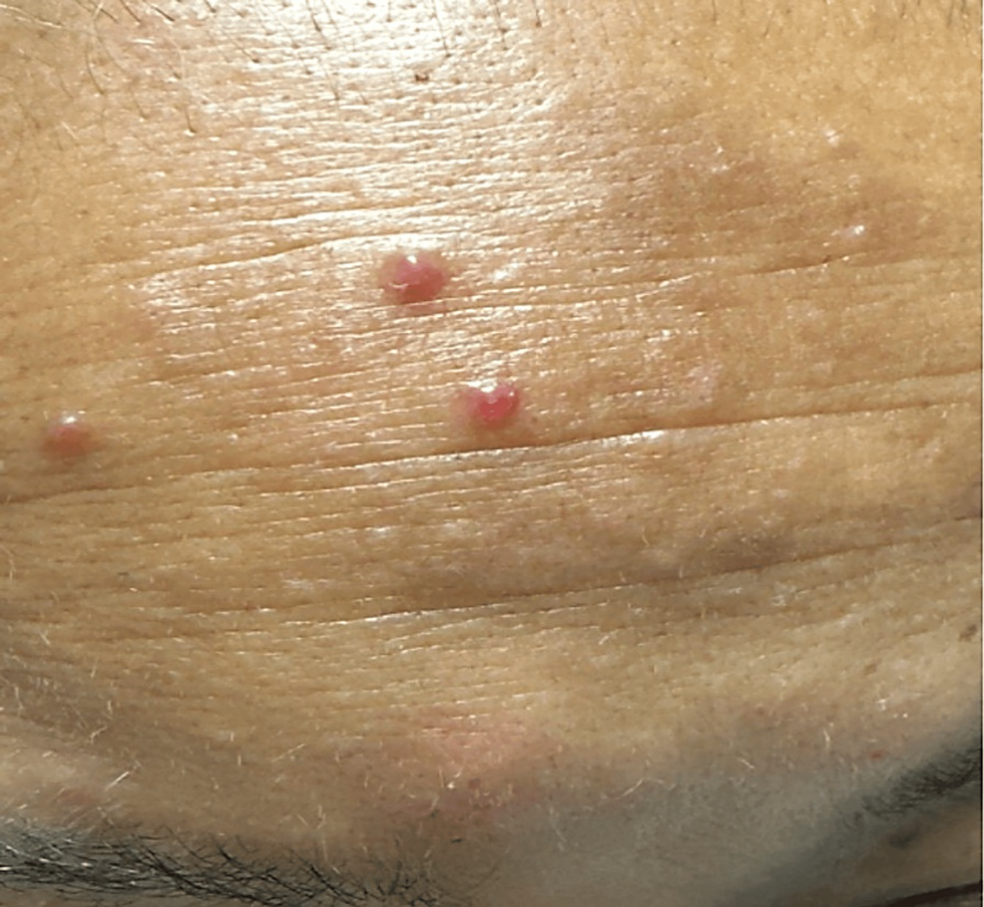
Shiny, red-to-pink dome-shaped papules scattered on the forehead

**Figure 2:**
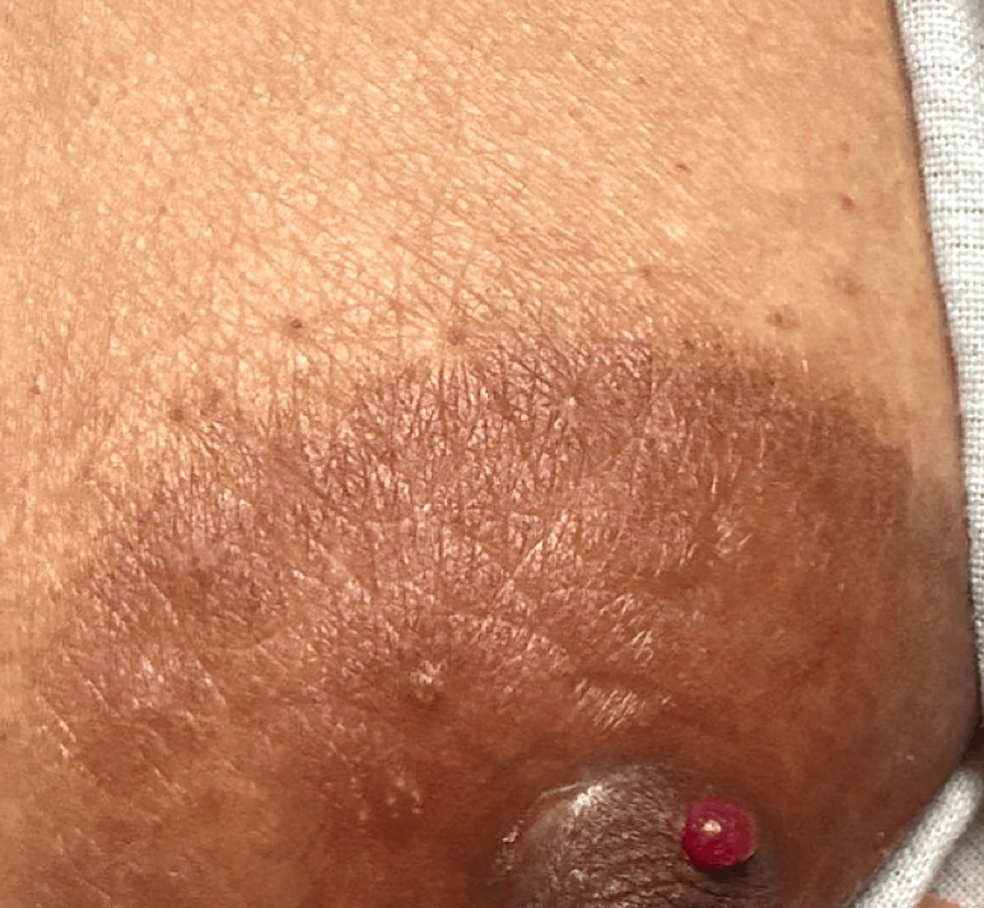
One shiny, bright-red dome-shaped papule on the nipple

**Figure 3:**
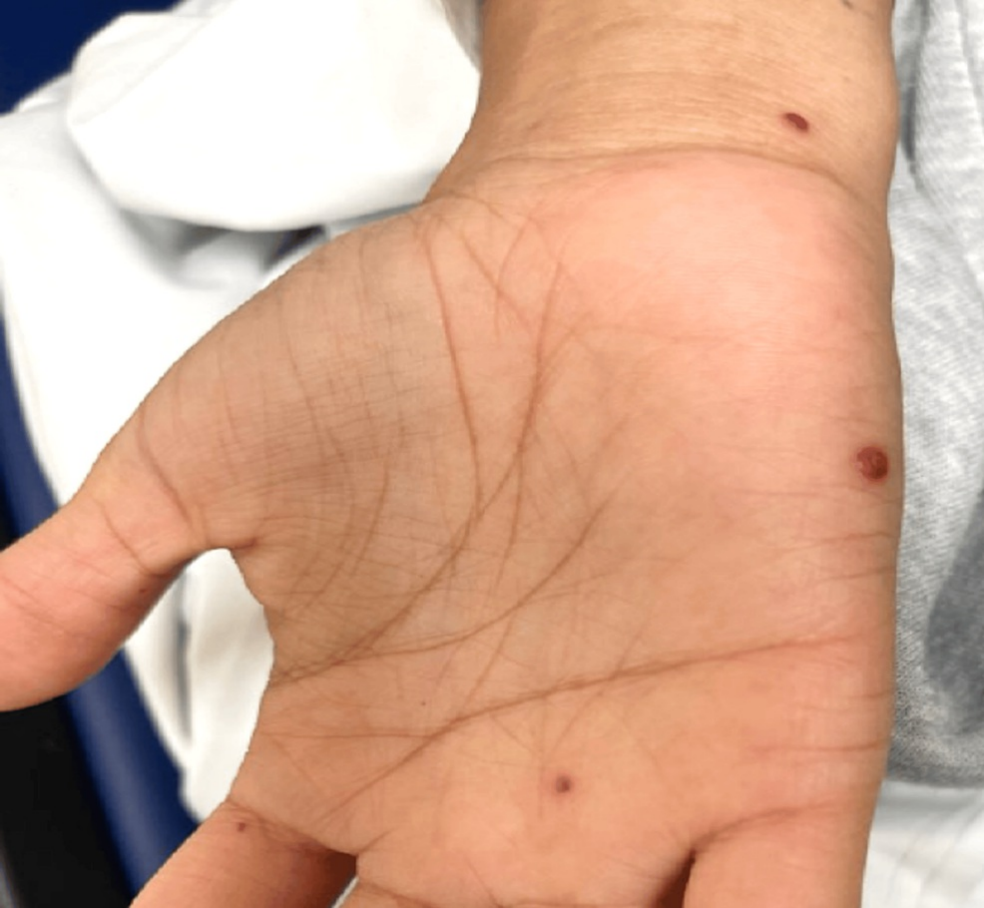
Deep-red, dome-shaped papules on the medial forearm, hand, palm, and second digit

**Figure 4:**
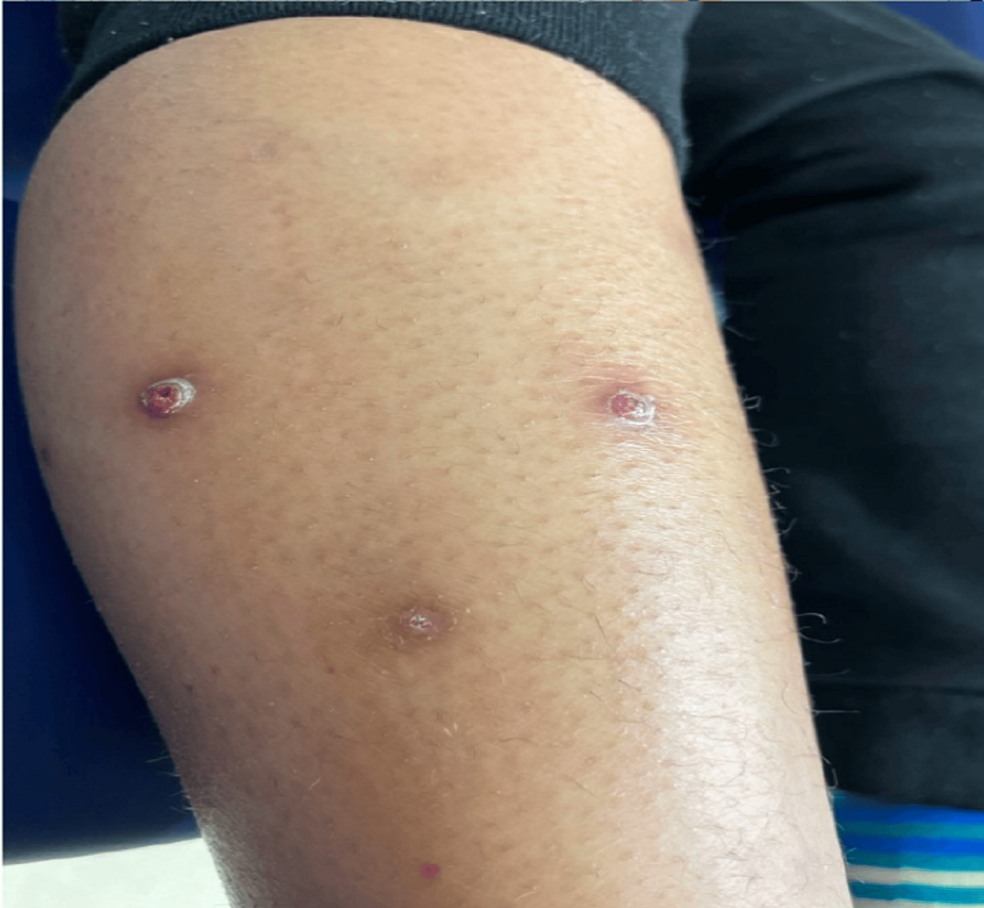
Pink to dark-brown hyperpigmented papules with prominent collarettes of scale on the lower leg

However, she re-presented due to epistaxis, nausea, vomiting, recurrent fevers, and a progressing rash. She noted the progression of bright-red, non-tender, non-pruritic, dome-shaped shiny papular lesions across her body (Figures 1-4). At this time, her CD4 count was 77/mm^3^ with a viral load of 39 copies/mL. The patient denied recent international travel, contact with sick individuals, and household pets. Although she intermittently interacted with a family member's cat, she did not pet the cat and did not sustain any scratches.

**Figure 5:**
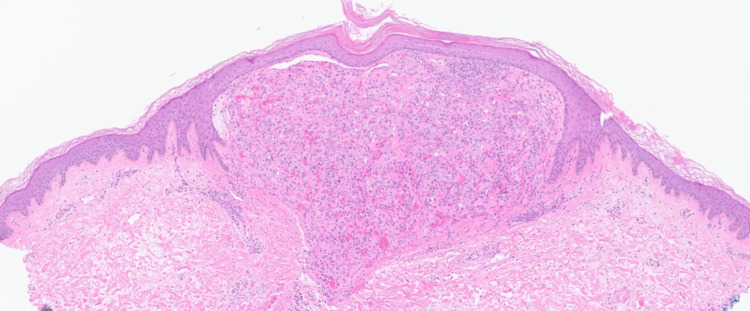
Low-power hematoxylin and eosin (H&E) staining showing a popular lesion composed of a well-defined dermal proliferation of vascular spaces

**Figure 6:**
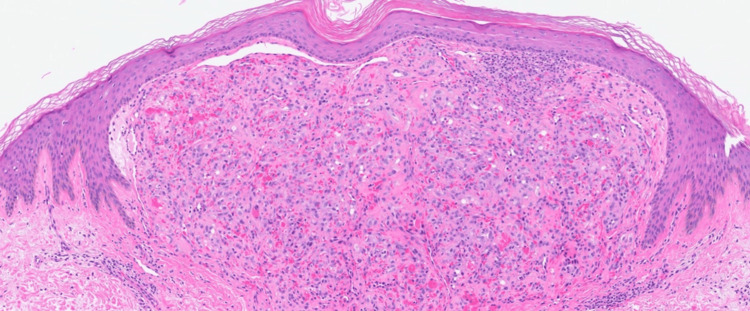
Higher-power hematoxylin and eosin (H&E) staining showing multiple vascular spaces with plump endothelial cells and collections of neutrophils

**Figure 7:**
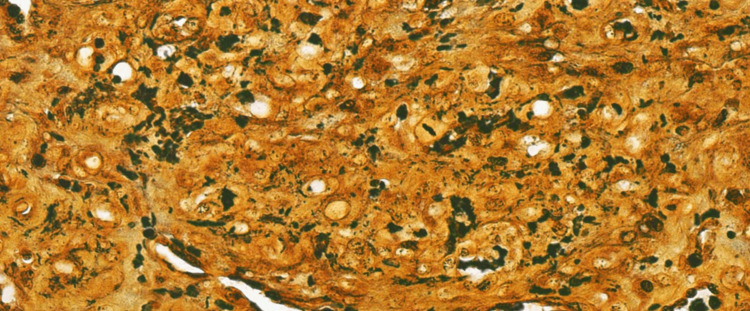
The Warthin-Starry stain is positive, demonstrating the darkly staining bacilli of Bartonella henselae

A punch biopsy of the right thigh was obtained and sent for histology and molecular testing for a definitive diagnosis. The histopathologic examination demonstrated a well-defined intradermal proliferation of vascular spaces with plump endothelial cells and scattered neutrophils surrounded by a collarette of normal epidermis on hematoxylin and eosin staining (Figures 5, 6). The Warthin-Starry stain revealed the presence of small, darkly staining, rod-shaped organisms presenting singularly and in clusters (Figure 7). Molecular analysis using 16S rDNA PCR identified* B. henselae* as the causative organism. The immunohistochemical staining for HHV8 was negative. Positive nonspecific staining was observed for both spirochete and Grocott methenamine silver stains. The patient started BA treatment with doxycycline 100 mg every 12 hours with an estimated course of approximately three months.

Another workup revealed MAC in acid-fast bacteria (AFB) blood cultures collected two months prior to her last hospitalization. She started treatment with azithromycin 500 mg daily, rifabutin 300 mg daily, and ethambutol 800 mg daily with plans to discontinue rifabutin after three weeks and continue dual agent therapy if fevers were controlled. ART was adjusted to dolutegravir and emtricitabine/tenofovir disoproxil fumarate instead of prior bictegravir/emtricitabine/tenofovir alafenamide to minimize drug-drug interactions. Follow-up appointments were scheduled for one to two weeks with primary care and the HIV community clinic. Unfortunately, there is no documentation indicating the patient attended these appointments.

## Discussion

First observed in the 1980s in individuals with AIDS, BA is a neovascular proliferative disorder due to infection with *B. henselae* and *B. quintana* [2]. BA primarily manifests in immunocompromised individuals, especially those with HIV/AIDS. However, in the era of effective ART, BA has become exceedingly rare, predominantly presenting in patients with CD4 counts less than 100/mm^3^ [12].

Approximately 1.2 million people are living with HIV in the United States, and it is estimated that 13% of these individuals are unaware of their current infectious status [10,13]. Of those who are aware of their disease, only 34% are adequately virally suppressed, and only 58% are engaged in continuous care [10]. Considering that a substantial portion of individuals living with HIV do not receive adequate treatment or follow-up, maintaining a broad differential diagnosis, including the most uncommon opportunistic infections, is imperative when caring for immunosuppressed patients.

While rarely encountered today, BA remains an important consideration of cutaneous and systemic opportunistic infection in individuals with HIV/AIDS given its subacute and insidious nature [14]. Its presentation can often be confounded by coinfection with other opportunistic microorganisms that are also present at low CD4 levels, leading to a delayed diagnosis. These opportunistic infections include coccidioidomycosis, PJP, histoplasmosis, cryptococcosis, toxoplasmosis, and, in the case of our patient, MAC [15].

To our knowledge, only two cases of BA with disseminated MAC have been reported in the literature [6,16]. Moreover, a study of 382 HIV-positive patients further demonstrates this rare co-occurrence, with *Bartonella* spp. and MAC isolated from blood cultures in only 1% of patients [7]. Both disseminated MAC and BA can present with nonspecific symptoms, which may overlap with one another and other opportunistic infections. Disseminated MAC or BA can present with fever, night sweats, weight loss, abdominal pain, lymphadenopathy, anemia, hepatosplenomegaly, and elevated alkaline phosphatase levels [1,2,17,18].

In addition to the nonspecific clinical presentation, distinguishing cutaneous BA from other benign and malignant lesions can be challenging. The characteristic smooth, dome-shaped, angiomatous papules and nodules of BA can resemble the malignant neoplastic, red to violaceous nodular lesions of KS [1]. The friable, bright-red papules and nodules of PG mimic BA, and both lesions may appear with a peripheral collarette of scales [1]. Histologically, PG closely resembles BA, showing lobular proliferation of capillaries with inflammatory infiltrate, which requires special stains, such as the Warthin-Starry stain, and molecular analysis to readily identify *Bartonella *spp. [1,2,17].

*Bartonella *spp. are susceptible to a wide range of agents, including penicillins, cephalosporins, aminoglycosides, chloramphenicol, tetracyclines, macrolides, rifampin, fluoroquinolones, and cotrimoxazole. However, only aminoglycosides have a bactericidal effect [19]. Although spontaneous resolution has been documented, the mainstays of treatment for BA include macrolide and tetracycline antibiotics [1]. Treatment with erythromycin or doxycycline has proven efficacious; however, azithromycin or clarithromycin may be used as alternatives [1,17]. In patients with contraindications to macrolides such as liver disease, doxycycline is recommended [19]. The minimum inhibitory concentrations correlate poorly with the in vivo efficacies of antimicrobial drugs in patients with *B. quintana* infection, and this discrepancy may be explained by the lack of bactericidal effect of most compounds and by sequestration of the bacterium in erythrocytes [19]. Additional antibiotic coverage may be necessary to eradicate the bacteria in these cases, such as gentamicin plus doxycycline [20]. Prompt initiation and maintenance of ART in addition to appropriate antibiotic prophylaxis, when necessary, remains the best strategy for the prevention of opportunistic infections in HIV-positive patients.

## Conclusions

In summary, BA is an exceedingly rare but treatable angioproliferative opportunistic infection in the era of effective ART. It predominantly manifests in severely immunocompromised individuals, especially those with HIV/AIDS. Many individuals living with HIV remain treatment-naive due to various reasons, resulting in susceptibility to numerous opportunistic infections such as BA. Ultimately, this highlights the importance of comprehensive evaluation, including dermatologic consultation for skin biopsy, when immunocompromised patients present with fevers of unknown origin and vascular-like lesions. Because one or multiple opportunistic infections often present with an insidious course and nonspecific symptoms, physicians must maintain a high index of suspicion in immunosuppressed patients to avoid delays in care.

## References

[1] Krishnan K, Calame A, Cockerell CJ (2013). Bacterial and atypical mycobacterial infections. Cutaneous Manifestations of HIV Disease.

[2] Cotell SL, Noskin GA (1994). Bacillary angiomatosis. Clinical and histologic features, diagnosis, and treatment. Arch Intern Med.

[3] Dehio C (2005). Bartonella-host-cell interactions and vascular tumour formation. Nat Rev Microbiol.

[4] Maguina C, Garcia PJ, Gotuzzo E, Cordero L, Spach DH (2001). Bartonellosis (Carrión's disease) in the modern era. Clin Infect Dis.

[5] Schwartz RA, Nychay SG, Janniger CK, Lambert WC (1997). Bacillary angiomatosis: presentation of six patients, some with unusual features. Br J Dermatol.

[6] Rovery C, Rolain JM, Lepidi H, Zandotti C, Moreau J, Brouqui P (2006). Bartonella quintana coinfection with Mycobacterium avium complex and CMV in an AIDS patient: case presentation. BMC Infect Dis.

[7] Koehler JE, Sanchez MA, Garrido CS (1997). Molecular epidemiology of Bartonella infections in patients with bacillary angiomatosis-peliosis. N Engl J Med.

[8] Gutiérrez R, Vayssier-Taussat M, Buffet JP, Harrus S (2017). Guidelines for the isolation, molecular detection, and characterization of Bartonella species. Vector Borne Zoonotic Dis.

[9] Shapira L, Rasis M, Binsky Ehrenreich I (2021). Laboratory diagnosis of 37 cases of Bartonella endocarditis based on enzyme immunoassay and real-time PCR. J Clin Microbiol.

[10] (2024). Guidelines for the prevention and treatment of opportunistic infections in adults and adolescents with HIV. https://clinicalinfo.hiv.gov/en/guidelines/hiv-clinical-guidelines-adult-and-adolescent-opportunistic-infections/introduction?view=full.

[11] Agrawal S, Singal A, Arora VK (2022). Bacillary angiomatosis in an immunocompetent patient: an unusual occurrence. Indian Dermatol Online J.

[12] Jung AC, Paauw DS (1998). Diagnosing HIV-related disease: using the CD4 count as a guide. J Gen Intern Med.

[13] Bosh KA, Hall HI, Eastham L, Daskalakis DC, Mermin JH (2021). Estimated annual number of HIV infections ─ United States, 1981-2019. MMWR Morb Mortal Wkly Rep.

[14] Schwartzman WA (1992). Infections due to Rochalimaea: the expanding clinical spectrum. Clin Infect Dis.

[15] Justiz Vaillant AA, Naik R (2023). HIV-1 associated opportunistic infections. StatPearls [Internet].

[16] Sagerman PM, Relman DA, Niroomand F, Niedt GW (1992). Localization of mycobacterium avium-intracellulare within a skin lesion of bacillary angiomatosis in a patient with AIDS. Diagn Mol Pathol.

[17] Akram SM, Anwar MY, Thandra KC, Rawla P (2023). Bacillary angiomatosis. StatPearls [Internet].

[18] Akram SM, Attia FN (2023). Mycobacterium avium complex. StatPearls [Internet].

[19] Foucault C, Brouqui P, Raoult D (2006). Bartonella quintana characteristics and clinical management. Emerg Infect Dis.

[20] Rolain JM, Brouqui P, Koehler JE, Maguina C, Dolan MJ, Raoult D (2004). Recommendations for treatment of human infections caused by Bartonella species. Antimicrob Agents Chemother.

